# Oral health-related quality of life in patients receiving dental treatment from dental therapists

**DOI:** 10.1186/s12955-026-02572-2

**Published:** 2026-07-21

**Authors:** Emma Louwagie, Michelle C. Arnett, Aparna Ingleshwar, Mike T. John, Danna R. Paulson, Yvette G. Reibel, Phonsuda Chanthavisouk

**Affiliations:** 1 Division of Dental Therapy, Department of Primary Dental Care, School of Dentistry, University of Minnesota, Minneapolis, 55455 USA; 2 Division of Dental Hygiene, Department of Primary Dental Care, School of Dentistry, University of Minnesota, Minneapolis, USA; 3 Department of Diagnostic & Biological Sciences, School of Dentistry, University of Minnesota, Minneapolis, USA

**Keywords:** Health-related quality of life, Oral health, Patient-reported outcomes, Quality of life, Dental hygiene, Dental therapy

## Abstract

**Purpose:**

Oral health-related quality of life (OHRQoL) measured by the 5-item Oral Health Impact Profile (OHIP-5) allows characterization of patients’ oral health impact into normative values in a university setting. This study aims to derive OHIP-5 normative values for patients seeking care from the dual degree student provider of the dental hygienist and dental therapist in Minnesota.

**Methods:**

Normative OHIP-5 values were determined in *n* = 2,835 patients at the University of Minnesota School of Dentistry, representing sample-based reference distributions. Summary scores were analysed using an empirical cumulative distribution function, with the 90th percentile threshold indicating typical versus elevated impact, while dimensional scores (Oral Function, Orofacial Pain, Orofacial Appearance, and Psychosocial Impact) assessed as elevated impact were considered frequent item response categories “fairly often” or “very often”.

**Results:**

The vast majority (90%) of patients seeking care from the dual degree student providers presented an OHIP-5 score of 6 or less, characterizing OHIP-5 scores of 7–20 points as “non-typically elevated”. The “non-typically elevated” dimension scores occurred in 7% of patients for Oral Function, 6% for Orofacial Pain, 11% for Orofacial Appearance, and 2% for Psychosocial Impacts.

**Conclusion:**

Understanding normative OHIP-5 values in patient populations treated by dual degree student providers in dental school settings supports clinical decision-making by guiding diagnostic, referral, and treatment pathways. Their role in preventive and restorative care advances patient-centered outcomes, aligning with the quadruple aim of healthcare.

## Introduction

Oral health-related quality of life (OHRQoL) describes a patient’s perception of the impact of their oral health [1]. OHRQoL is four-dimensional to encompass the patient’s perception of their Oral Function, Orofacial Pain, Orofacial Appearance, and Psychosocial Impact [2]. Patient-reported outcomes (PROs) are a patient’s self-report of their personal health, without the interpretation of a health professional [3]. Dental patient-reported outcomes (dPROs) are the ideal outcome to determine the patient’s oral health perspective [4]. The Oral Health Impact Profile (OHIP) is an instrument that measures dPROs to determine oral health impact and can be utilized to determine patients’ perceived response to dental treatment [5]. 

OHIP is one of the most widely used instruments to measure OHRQoL in dentistry [6]. OHIP assesses an individual’s perception of how oral health affects their quality of life [5]. The shortest version of this instrument is the OHIP-5, which includes five items that illustrate the four dimensions of OHRQoL [2, 7]. It is crucial for dental providers to understand and measure OHRQoL as this provides support to directly address patients’ primary concerns and plan treatments effectively. In addition to considering the clinician’s evaluation of the patient (checking gum health, cavity, and other oral health evaluations), assessing the patient’s perspective through dPROs to identify the general adult population normative value is a critical point in patient-centered care. The OHIP-5 is a reliable and valid instrument that captures 90% of the information captured by the original version of OHIP, which has 49-items [5, 7, 8]. The short form provides ease in time and effort from the provider and patient’s perspective.

It is important to measure OHRQoL to explore the impact that dental treatments have on patients’ lives. Chanthavisouk et al. recently identified 13 distinct patient populations that seek dental care from dual-degree providers, the dental hygienist and dental therapists (DTs), highlighting the lack of evidence on OHRQoL for these distinct patient populations [9]. Dental therapy is a relatively new profession in the United States (US) with limited research on the impact of treatments on these distinct patient populations’ OHRQoL. The profession was introduced to the oral health care team with the intention of expanding access to dental care, especially for children and patients in long-term care facilities [10, 11]. By 2009, the state of Minnesota authorized the training and implementation of DTs to provide dental care in underserved communities [10]. In the US, the scope of practice of DTs includes dental examinations, diagnosis and treatment planning, exposing and interpreting radiographs, restorative functions of caries lesions, including amalgam, composite, and preformed stainless-steel crowns, pulpotomies, and the extraction of primary teeth, along with permanent teeth; all dependent on state legislation and/or certification as an advanced dental therapist [12, 13]. In the US, some DTs receive their education in dental hygiene [9, 14]. In addition to oral health education, preventative services such as fluoride therapy, pit and fissure sealants, and dietary counselling, as a dental hygienist, the scope of practice spans to include prophylaxis and periodontal treatments such as scaling and root planing [10, 15, 16]. 

The scope of practice for the DTs allows these providers to focus on effective and quality dental care in pediatric and adolescent populations [10]. However, there is currently no evidence on adult patients’ OHRQoL measured by dPROs. It is imperative to provide normative values on the impact of adult patients’ OHRQoL measures by dPROs in distinct patient populations for dual-degree providers. The normative value is a population-based reference score that represents the typical or expected level of OHRQoL within a defined group. It allows individual or subgroup OHIP scores to be interpreted relative to what is considered standard for the general population or comparable patient populations. Normative values help determine whether a patient’s OHRQoL impact is lower, typical, or higher than what is normally observed. Conceptually, the 90th percentile was selected to encompass the majority of the studied population, the general adult population [17]. The normative values can help with patient-centered treatment decision-making. This research aims to characterize normative values in OHRQoL in adult patient populations seeking dental treatment from dual-degree students (the dental hygienist and dental therapist) in a university setting.

## Methods

This cross-sectional, single-site study was approved and exempted from human subjects’ research with no greater than minimal risk by the University of Minnesota (UMN) Institutional Review Board (IRB) (STUDY00024185). As this study was exempt from human subject research, a clinical trial number is not applicable. The study was conducted in accordance with US federal standards for the protection of human subjects (45 CFR 46) [18]. 

### Study population

The study included adult patients at the UMN School of Dentistry (SOD) who received dental care from dual-degree student providers. Dental patients enrolled in UMN SOD clinics are provided with a general consent form that includes a research consent. Patients were included in the study if they opted in to the research consent. Eligibility criteria included adult patients (18 years or older) who received care from a dual-degree student provider between January 2018 and November 2024, with consent for research stored in the axiUm electronic health record (EHR) and completed OHIP-5 at their visit. De-identified data was accessed for research purposes on September 6th, 2024. A total of *n* = 3,228 patients received dental care from dual-degree student providers during this period. Due to the inclusion criteria requiring the completion of OHIP-5, missing data were identified as unanswered items within the 5-item survey.

### Outcome assessment

The 5-item OHIP questionnaire is collected as part of routine dental care and stored in the axiUm medical history for all patients at the UMN SOD. The baseline OHIP-5 assesses overall OHRQoL, with a total score calculated from all items, and dimension scores calculated from one item each that serve as indicators for the four dimensions [19]. Baseline OHIP-5 was collected before the start of the treatment and required completion of treatment on the same day. As dental hygiene and dental therapy treatments are more focused on prevention, in-person follow-up data collection is difficult, as many patients do not return once treatment is complete within a shorter time frame, for example, within the following week. Once the treatment is complete, patients are normally scheduled for a recall appointment further along than the necessary follow-up data collection window. Therefore, in this study, only baseline OHIP-5 was included. Although the dimension of Oral Function is captured by two items, “chewing difficulty” and “less flavor in food”, the former is considered the primary Oral Function indicator compared to the latter; hence, it is used as the indicator of choice for the Oral Function dimension in this study [7, 17]. A reference period of the last 7 days was provided for the patient to answer the OHIP-5 questionnaire [20]. The patient was provided with the response options on a 5-point ordinal scale (“never” to “very often”), which are summed to result in a total OHIP-5 summary score ranging from 0 to 20, whereas dimension scores range from 0 to 4.

### Data analysis

Means and standard deviations were calculated for OHIP-5 summary scores (representing overall OHRQoL) and for the dimension scores (representing dimensional OHRQoL impairment). A 95% confidence interval was also calculated for the summary score mean. A box plot displaying the median, interquartile range, and overall range was created to characterize the central tendency and spread of the summary scores. The overall distribution of summary scores was further illustrated using an empirical cumulative distribution function. The 90th percentile was identified as a cut-off point to differentiate between “typical” and “non-typically elevated” OHRQoL impact. The 90th percentile was used to reflect the upper threshold of OHRQoL impact for the majority of patients as a meaningful indicator of population-level oral health burden [17]. Normative values for each dimension were displayed using bar charts. A dimension was classified as having a “non-typically elevated” impact as a distribution-based threshold rather than a clinical cutoff if participants reported experiencing a problem “fairly often” or “very often.” Due to the right-skewed distribution of data, the distributional statistics were provided for the four dimensions of OHRQoL. Analyses were performed using Stata version 18 (StataCorp, 2023) [21]. 

## Results

A total of *n* = 7,401 OHIP-5 questionnaires were collected between January 2018 and September 2024. Of the total OHIP-5 questionnaires collected, *n* = 6,982 had zero or only one missing answer, with an item response rate of 97%. The OHIP-5 questionnaires were collected from a total of *n* = 3,228 individual patients. After imputing missing response data and retaining the baseline OHIP-5 response per patient, *n* = 2,835 OHIP-5 questionnaires were utilized in the final data analysis. The sociodemographic data collected include age, gender, race, and insurance type, if present, as seen in Table 1. The response rate of sociodemographic data varied for individual data points, as some questions were optional responses, such as race, ethnicity, and gender. These variables present as missing data due to the nature of the optional response.

**Table 1:** Sociodemographic data of patients receiving treatment from a dental hygiene and dental therapist student provider

Patient Characteristics (*n* = 2,835)		*n*(%)
Age [Mean/(SD)]	53.44 (21.51)	
Gender*		
Female	1,455 (51.3%)	
Male	1,377 (48.6%)	
Transgender	2 (0.07%)	
Race*		
White	1,501 (52.9%)	
Black	340 (12.0%)	
Asian	113 (4.0%)	
Native	12 (0.4%)	
Middle eastern/North African	6 (0.2%)	
Pacific Islander	2 (0.1%)	
Other/Multiple race	226 (8.0%)	
Ethnicity*		
Hispanic/Latino	190 (6.7%)	
Not Hispanic/Latino	1,990 (70.2%)	
Insurance Type		
Private	1,076 (38.0%)	
Private/Public	183 (6.5%)	
Public	1,085 (38.3%)	
Uninsured	491 (17.3%)	

*Proportions may not sum up to 100 due to missing information

**Fig. 1:**
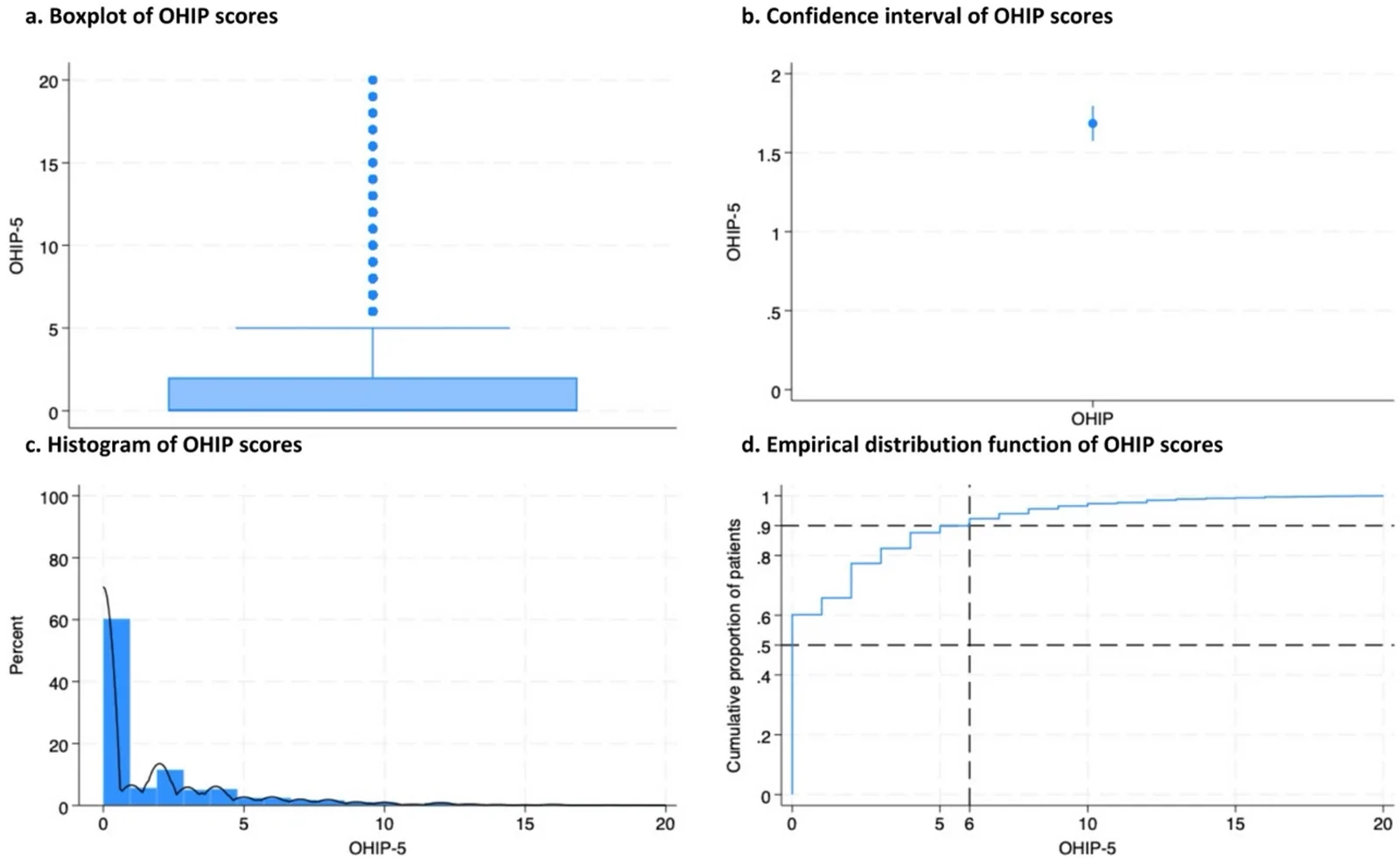
**a** - **d**. Oral health-related quality of life in patients receiving care from dental hygiene and dental therapist student providers

The distribution of OHIP-5 summary scores in patients receiving treatment from a dual-degree student provider can be seen in Fig. 1a. The mean OHIP score was 1.68 with a standard deviation of 3.01. The mean OHIP-5 summary score with 95% confidence interval is depicted in Fig. 1b; the narrow confidence interval lends support to the precision of this estimate. The frequency distribution of OHIP-5 summary scores (Fig. 1c) shows that roughly 60% of the patients in the study have no oral health impact, i.e., OHIP-5 summary score is 0. The empirical cumulative distribution for patients receiving care from the dental therapist is seen in Fig. 1d, stating that 90% of patients receive a score of 6 or less.

**Figs. 2:**
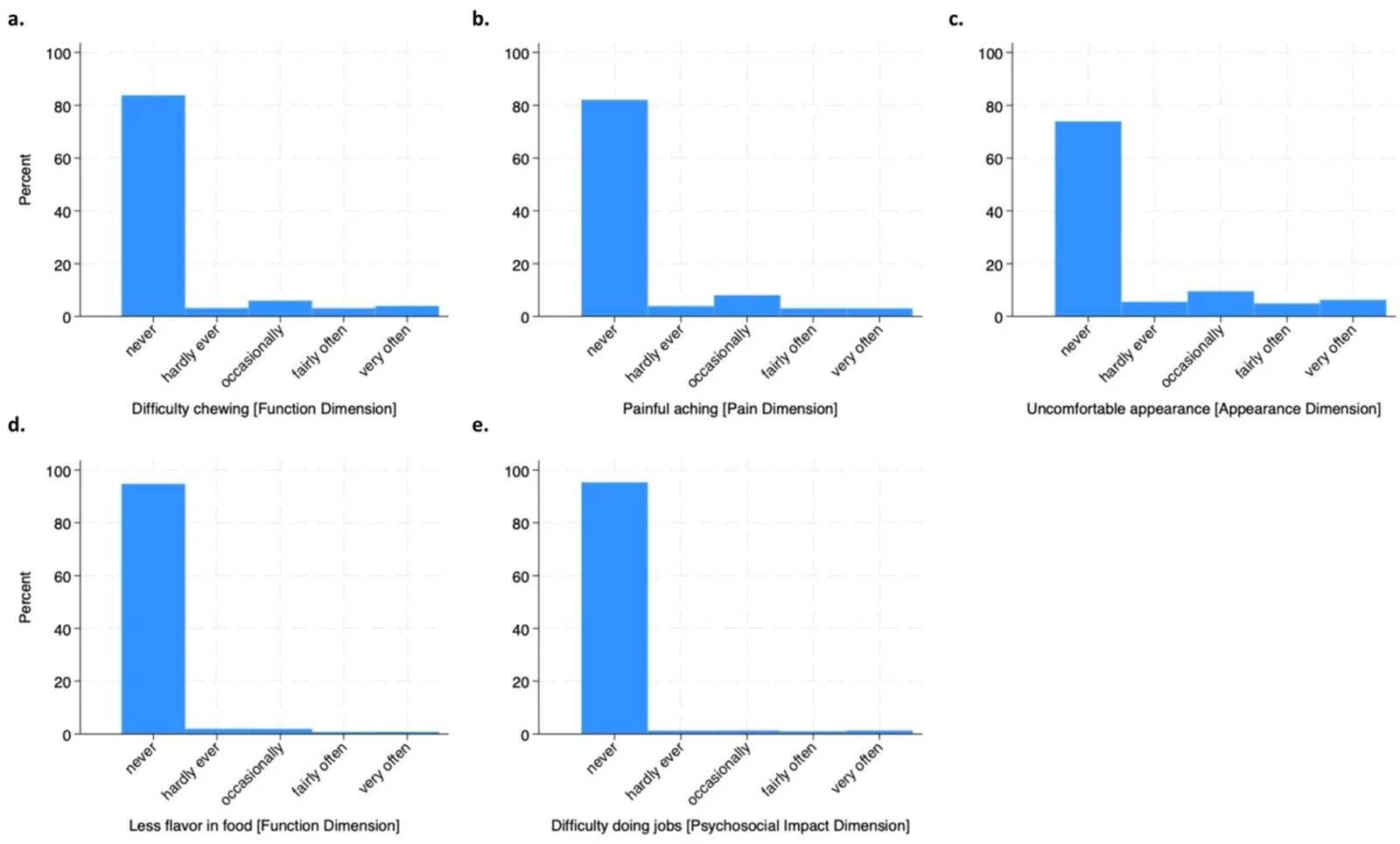
**a** - **e**. Percentage distribution of four-dimensional impact among patients receiving care from a dental hygiene and dental therapist student provider

Each item from OHIP-5 was analysed to provide insight into the four-dimensional OHRQoL impact scores. Figure 2a through 2e illustrate the percentage distribution of recorded item responses. Figure 2a shows responses for question one of OHIP-5, which belongs to the Oral Function dimension, with a mean of 0.40 and a standard deviation of 1.01. Figure 2b presents question two from the Orofacial Pain dimension, scoring a mean of 0.41 with a standard deviation (SD) of 0.97. Figure 2c shows question three, representing the Orofacial Appearance dimension, with a score of 0.64(1.21). Figure 2d illustrates question four, the second item under the Oral Function dimension, scoring 0.11(0.52). Finally, Fig. 2e represents question five, covering the psychosocial impact dimension, with a score of 0.12(0.58). In conclusion, the “non-typically elevated” dimension scores occurred in 7% of patients for Oral Function, 6% for Orofacial Pain, 11% for Orofacial Appearance, and 2% for Psychosocial Impacts.

## Discussion

This is the first study known to the authors to investigate OHRQoL and identify the normative values for the adult patient population who seek dental care from dual degree (dental hygiene and dental therapy) student providers. The data depict that most patients (90%) treated by the dual degree student providers have an OHRQoL impact score of 6 or less. As this study provides cross-sectional and descriptive data, the interpretations from the results are limited to the characterization of OHIP-5 score distributions for dental hygiene and dental therapy student providers in a university setting.

### Comparison with the literature

There is limited research on the OHRQoL of patients who seek care from the dual-degree student provider available for comparison. It is important to acknowledge that more generalized studies need to be completed for this distinct patient population. Flavin et al. determined the baseline OHRQoL score of 11.14 for adults receiving inpatient psychiatric care who were seen by a hospital dental hygienist [22]. This study’s university setting took place in a medical facility, in comparison to the current study (a dental school) [22]. The OHRQoL score in this study is considerably lower, with a baseline of 6 or less. However, Flavin. et al., study had a smaller sample size of 24 patients, with more females than males, with no demographic information related to insurance type or residence [22]. The sample size *n* = 2,835 in this study provides a larger sample size, along with an equal distribution of genders. Another consideration is that this study assesses the general adult population, whereas Flavin et al. retrieved data from adults currently receiving inpatient psychiatric care in a hospital setting [22]. The differences in score could be due to the type of population studied (inpatient psychiatric care versus the general adult population). Future research should look at different patient populations in a variety of settings. Clinically, the sample-based normative values of this study can be applied to aid in patient-centered treatment decision-making for dual-degree student providers. With a potential baseline OHRQoL score of 6 or less, this gives dual-degree student providers a general insight into the normal limits.

In patients who seek care from the dual degree student providers who present with an OHRQoL score greater than 7, the clinician should be investigating one or more aspects of the four dimensions, i.e., Orofacial Function, Orofacial Pain, Orofacial Appearance, and Psychosocial Impact. Upon further investigation, the dual-degree student provider can utilize the sample-based normative values to determine if the patient’s dental issue is considered within normal limits or to aid in treatment decision-making. For example, a patient reports a high Orofacial Pain score and recently received a space maintainer. The completion of baseline OHIP-5 before an exam allows the provider to review the OHIP-5 responses before entering the room. They can quickly confirm the patient’s perspective and verify during the exam that the pain is coming from the new appliance. The dental hygienist and dental therapist can provide the patient with the sample-based normative value as a measure to better communicate and reassure the patient of the expected OHRQoL impact for that dental treatment. This can be utilized in the treatment planning phase as well to inform patients what they can expect once they have completed said treatment.

### Exploratory clinical implications

The exploratory results of this study can also be applied to the dual degree student providers (dental hygienist and dental therapist) role and impact in the dental field, or as a form of medical-dental integration. The results show the value that dual-degree student providers have on patients’ treatment and the potential for identification of the baseline impact of their OHRQoL in a university setting [10, 15, 16]. As mentioned previously, DTs were created to expand access to care for underserved populations [10]. Studies have shown that DTs provide high-quality dental care with an absence of negative effects on oral health outcomes, with positive impacts on access to dental care and cost [23]. Research on the impact on patients’ OHRQoL by licensed dental therapists should be assessed. If a patient presents with an OHRQoL score of 7 or higher, it can signal the student provider to adjust or tailor the day’s treatment plan and guide a conversation about potential changes in care. In a medical-dental integration example, Flavin et al. described a baseline OHIP-5 score of 11.17 for adults receiving inpatient psychiatric care [22]. Future medical offices, such as a primary care provider, could utilize OHIP-5 as part of the intake form to note whether the patient may need a referral to the nearest dental office. Assessment in the presence of dual-degree dental hygiene and dental therapy providers in the dental team can further determine the impact of dental patients’ OHRQoL. This further assists in medical-dental integration models to expand access to oral health care.

## Strengths and limitations

Strengths in this study include a high confidence interval of 95%, along with a large sample size of *n* = 2,835. A limitation of the study is that the patient population was located at the UMN SOD. More studies in different clinical settings, such as Federally Qualified Healthcare Centers (FQHCs), community clinics, and other dental clinic settings, should be completed to provide more generalized data among different practice types. More OHIP scores need to be gathered for minority populations to allow for a more generalizable data set for OHRQoL based on race and ethnicity. There needs to be more research on minority populations. This need is largely due to the study’s majority population based being white females and males, specific to Minnesota. This study only included baseline OHIP-5 data. Future studies should investigate an automated online system to collect follow-up data, due to the nature of dental hygiene and dental therapy treatments requiring minimal in-person follow-up appointments. An automated online system would allow for a standardized protocol as well as reduce the burden on the in-person clinic flow.

## Conclusion

The sample-based normative values for OHIP-5 scores of underserved populations in the dental hygiene and dental therapy scope of practice are needed for patients, clinicians, researchers, and other stakeholders to understand patients’ OHRQoL impact. This study determined that in most of the population (90%), the mean OHRQoL score of patients who received care from dual degree student providers was determined to be 6 or less. The normative values can be applied clinically to aid in patient-centered treatment decision-making when the OHRQoL score is greater than 6, upon completion of an in-depth investigation of the four dimensions of OHRQoL.

## Data Availability

The datasets used and/or analysed during the current study are available from the corresponding author on reasonable request.
